# Dropout, Nonusage Attrition, and Pretreatment Predictors of Nonusage Attrition in a Commercial Web-Based Weight Loss Program

**DOI:** 10.2196/jmir.1640

**Published:** 2010-12-14

**Authors:** Melinda J Neve, Clare E Collins, Philip J Morgan

**Affiliations:** ^2^School of EducationFaculty of Education and ArtsUniversity of NewcastleCallaghanAustralia; ^1^School of Health SciencesFaculty of HealthThe University of NewcastleCallaghanAustralia

**Keywords:** Weight loss, Internet, commercial sector, retention

## Abstract

**Background:**

An understanding of the factors that predict retention and website use are critical to the development of effective Web-based weight loss interventions. However, poor retention (dropout attrition) and website utilization (nonusage attrition) are major inhibitors to the effectiveness of Web-based programs.

**Objective:**

The study aimed to (1) describe the prevalence of dropout and nonusage attrition and (2) examine pretreatment predictors of nonusage attrition in a cohort of commercial Web-based weight loss program participants.

**Methods:**

Participants enrolled in the online program, The Biggest Loser Club, Australia, from August 15, 2007, to May 31, 2008. Only those who subscribed for 12 or 52 weeks were included in this study. All data were collected by the program proprietors, SP Health Co Pty Ltd (Sydney, Australia), and provided in “deidentified” form. Data collected included responses to a pretreatment survey (sociodemographic and behavioral characteristics), subscription history (date of enrollment and subscription end), and website use (log-ins, food and exercise diary entries, weigh-ins, and forum posts). Participants were classified as a member of the program at 12 or 52 weeks if they held an active subscription plan at that point in time. Participants were classified as nonusers at 12 or 52 weeks if they had stopped using all of the website features and had not returned. Predictors of nonusage attrition were explored using Cox proportional hazards regression analysis.

**Results:**

Of the 9599 eligible participants, 6943 (72%) subscribed to the program for 12 weeks, and 2656 (28%) subscribed for 52 weeks. Of all participants, 31% (2975/9599) were classified as overweight, 61% (5866/9599) were classified as obese, 86% (8279/9599) were female, and participants’ mean (SD) age was 35.7 (9.5) years. The 12 week and 52 week subscribers’ retention rates were 97% and 77% respectively. Of 12 week subscribers, 35% were classified as program “users” after 12 weeks, and 30% of 52 week subscribers were classified as “users” after 52 weeks. Significant predictors of nonusage attrition among 12 week subscribers included age (hazard ratio for 45 to 55 years of age = 0.83, 95% confidence interval [CI] 0.73 - 0.93, P = .001; hazard ratio for 55 to 65 years of age = 0.80, 95% CI 0.66 - 0.99, P = .04), exercise level (hazard ratio = 0.76, 95% CI 0.72 - 0.81, P < .001), emotional eating (hazard ratio = 1.11, 95% CI 1.04 - 1.18, P = .001), eating breakfast (hazard ratio = 0.88, 95% CI 0.82 - 0.95, P = .001), and skipping meals (hazard ratio = 1.12, 95% CI 1.04 -1.19, P = .001). For 52 week subscribers, eating breakfast (hazard ratio = 0.88, 95% CI 0.79 - 0.99, P = .04) and not drinking tea or coffee with sugar (hazard ratio = 1.23, 95% CI 1.11 - 1.37, P < .001) were the pretreatment characteristics that significantly decreased risk of nonusage attrition.

**Conclusions:**

The findings demonstrate a high prevalence of nonusage attrition among a cohort of commercial Web-based weight loss program participants. Several sociodemographic and behavioral factors were shown to independently predict nonusage attrition.

## Introduction

Public health interventions delivered via the Internet are becoming increasingly popular, and evidence to support their ability to achieve health-related behavior change and positive health outcomes is growing [1]. However, there is a need for Internet-delivered health and lifestyle interventions to minimize attrition and boost utilization rates in order to improve effectiveness [2-4]. A recent systematic review of Web-based weight loss interventions found that these interventions have the potential to achieve significant weight loss; however, they can also suffer from high dropout and poor utilization [5].

Retention rates published to date for Web-based weight loss programs range from 20% to 100%, with the majority less than 80% [5]. There may be an association between increased numbers of tasks prescribed or degree of participation required in Web-based interventions with lower retention rates. For example, studies comparing participants in Web-based weight loss interventions with control groups almost universally report higher retention among the control groups [6-12]. Furthermore, in some studies, where Web-based weight loss interventions are compared with Web-based interventions with a greater number of features, higher retentions rates are often found in the Web-based interventions with fewer features [13-16].

The majority of Web-based weight loss interventions report low website usage and experience a steady drop in usage over time [2]. Many participants also do not achieve the level of use prescribed by the program [2,17-19]. It appears, however, that the addition of evidence-based components to Web-based interventions such as behavioral therapy, human counseling, or motivational interviewing may result in greater website use compared with Web-based interventions that provide basic education or information only [14-16,20]. For example, studies have demonstrated significantly higher numbers of log-ins [14-16,20] as well as more self-monitoring occasions and higher attendance in online meetings [14] with the addition of these evidence-based components. Recent systematic reviews of Web-based weight loss interventions have also acknowledged the inverse relationship between website use and weight loss [4,18]. Therefore, the ability of Web-based interventions to maximize utilization and retain participants is a crucial component of efforts to enhance effectiveness.

As participants can potentially fail to drop out of Web-based interventions but stop using the website, Eysenbach [4] has suggested that exploration of attrition rates should include dropout attrition rates (ie, participants who do not complete the study/program) and nonusage attrition rates (ie, participants who stop using the website). Such knowledge is required to improve our understanding of how participants use Web-based programs. Eysenbach [4] has also highlighted the importance of exploring predictors of attrition in Web-based programs. Previous research has investigated pretreatment predictors of dropout attrition from weight loss interventions and demonstrated key sociodemographic characteristics (education level [21], employment status [21,22], age [23,24], gender [25]) and behavioral factors (number of previous weight loss attempts [21,26,27], dietary intake [26], emotional status [27,28], binge eating [28], and weight loss expectations [26]) that were predictive of dropout attrition. However, no consistent patterns of pretreatment predictors of nonusage attrition from Web-based weight loss interventions have been identified to date. Potential predictors include gender [13,29], age [13,29,30], motivation [13], body mass index [30], physical activity [30], and fruit and vegetable consumption [30].

To date, studies investigating Web-based weight loss programs have predominantly been randomized controlled trials (RCTs). However, RCTs could potentially overestimate or underestimate participant attrition and website use due to the inherent characteristics of volunteers and study rigor (eg, motivated participants, additional assessment sessions, subject retention strategies, greater accountability, and contact with study staff). Therefore, RCTs may not represent attrition or website usage in the “real world.” Studies that follow real-world participants of Web-based weight loss programs are, therefore, needed to ascertain true dropout and nonusage attrition rates in order to enhance program effectiveness.

Therefore, the first aim of this study was to describe in a large cohort of real-world users of a commercial Web-based weight loss program, the prevalence of dropout and nonusage attrition. The second aim was to determine which pretreatment sociodemographic and behavioral characteristics predict nonusage attrition.

## Methods

### Participants and Design

Participants were adults 18 to 75 years of age who enrolled in a commercial Web-based weight loss program from August 15, 2007, through May 31, 2008, and paid a subscription to access the program. A self-reported body mass index (BMI) of greater than or equal to 22 kg/m^2^ was required to enroll in the program. Only participants who subscribed for 12 or 52 weeks were included in this study, as they are the most predominant subscription lengths. Participants who did not pay for their initial subscription (eg, free promotional program trials) were excluded. Data related to free or nonconsecutive memberships (≥ 7 days apart) were also not included in the analysis. Membership status and website use were tracked for the duration of the subscription.

### The Commercial Web-Based Weight Loss Program

SP Health Co Pty Ltd (Sydney, Australia) developed the Web-based weight loss platform that is commercially available as The Biggest Loser Club. In summary, the online program incorporates key evidence-based weight management strategies and aligns with key elements of social cognitive theory [31] including self-management, social support, self-efficacy, outcome expectations and expectancies, and perceived barriers/facilitators. The key features of the program include goal setting (goal weight, daily calorie goal, and weekly exercise goals), self-monitoring of weight via weekly weigh-ins, as well as food and exercise using an online diary, educational material provided by weekly email, and an online discussion *forum*. Participants who enroll in the program purchase a specific subscription plan. The subscription plans are of 4, 12, 16, or 52 week’s duration and are either paid for prospectively at enrollment or by monthly installments. In 2007–2008 the cost of the program ranged from A$16.50 to A$79.95 per month. The cost per month to the participant was lower if they subscribed for longer and/or paid up front. Participants were predominantly recruited via marketing of the program through a reality television program, *The Biggest Loser, Australia*.

### Data Collection

The proprietors of program, SP Health Co, store all data entered by participants accessing the program website. Data stored include responses to an enrollment survey, subscription plans held, and use of a number of the website features (log-ins, online food and activity diary entries, weigh-ins, and posts to the discussion forum). SP Health Co extracted stored data in “deidentifiable” form for up to 52 weeks from enrollment for all participants who met the inclusion criteria. Ethics approval for the study was obtained from the University of Newcastle Human Research Ethics Committee.

### Pretreatment Characteristics

Participants’ pretreatment characteristics were captured from the enrollment survey. Participants’ self-reported height and weight were used to calculate BMI (weight in kilograms divided by height in meters squared), which was categorized as healthy, overweight, or obese using the World Health Organization’s BMI classification [32]. Reported postcodes were assigned an Index of Relative of Socioeconomic Advantage and Disadvantage (IRSAD) decile (ranked from 1 = disadvantaged to 10 = advantaged) as an indicator of socioeconomic status [33]. The remoteness of the area in which participants lived was classified according to the Accessibility/Remoteness Index of Australia (ARIA) of their postcode [34]. Participants’ reasons for wanting to achieve weight loss were grouped as health-related reasons (eg, doctor recommended or health scare) and reasons not related to health (eg, to look good or to enhance one’s love life), and participants were categorized as having 1 or more health-related reasons or no health-related reasons for wanting to lose weight. Participants also selected their reasons for eating (to ease emotional upset, for the joy of it, to reduce stress, and out of boredom), whether they had eating habits associated with weight gain (frying foods, using butter in cooking, drinking full sugar soft drinks, skipping meals, drinking tea or coffee with sugar, not eating breakfast, not using low fat products, keeping snack foods in the house, and not drinking 6 or more glasses of water a day) and the number of days they exercised per week. Age and gender were also captured from the enrollment survey.

### Website Use

Website use was assessed by summing available usage data. Participants were classified as having used the website on any given day if they logged in, made an entry in the diary, posted to the forum, and/or weighed in. The total number of days per 4 week period each participant “used” the website was calculated and categorized as 0 days, 1 to 3 days, 4 to 7 days, 8 to 15 days and 16 or more days. All website use variables were calculated from enrollment to 12 and 52 weeks for the 12- and 52 week subscribers respectively.

### Dropout Attrition

The date a participant enrolled in program and the date membership ceased were used to calculate the number of days each participant was a member of the program (ie, duration of membership). The date membership ceased was the end date of the participant’s subscription plan unless there were special circumstances that prevented the participant from completing the subscription (eg, pregnancy or financial constraints). Participants were classified as members of the program at 12 or 52 weeks if they held an active subscription plan at that point in time (≥ 78 days for 12 week subscriptions and ≥ 359 days for 52 week subscriptions). Otherwise they were classified as a dropout.

### Nonusage Attrition

Nonusage attrition was only considered for participants who completed their subscription (ie, they did not drop out). Participants were classified as a nonuser at 12 or 52 weeks if they stopped using the website features (ie, no log-ins, food/activity diary entries, weigh-ins, or posts to the discussion forum). The week a participant was classified as a nonuser was the week he or she ceased using the website and did not return.

### Data Analysis

Data analysis was undertaken using Stata 11 IC (StataCorp LP, College Station, USA). Participant pretreatment characteristics were described as means (SD) for continuous variables and percentage for categorical variables. Subscription length (12 and 52 weeks) group differences were tested using independent *t* tests for continuous variables and chi-square tests for categorical variables. Participants’ pretreatment characteristics were investigated as predictors of nonusage attrition for 12- and 52 week subscribers using Cox proportional hazards regression analyses. The time variable was the duration of usage (in weeks), and nonusage was considered a failure. Univariate analyses were conducted on all pretreatment predictor variables of interest and those with *P* < .2 were included in a stepwise regression analysis to find the most parsimonious model. The proportional hazards assumption was tested for each model using the Schoenfeld residuals. The significance level was set at alpha = .05.

## Results

### Pretreatment Characteristics and Website Use

**Figure 1:**
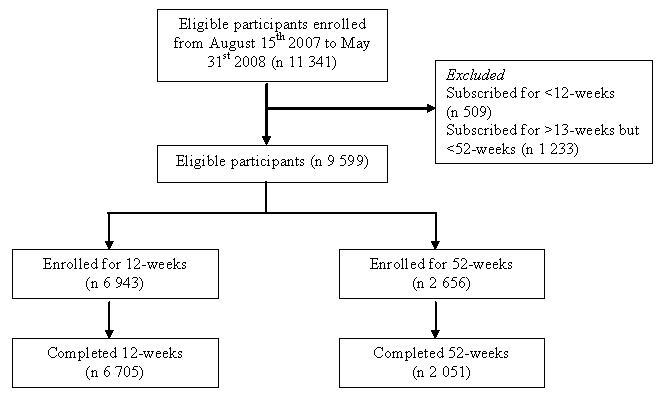
Participant flow

Of the 11,341 participants who enrolled in the commercial Web-based weight loss program between August 15, 2007, and May 31, 2008, 9599 were eligible for inclusion in the study, and 1742 were excluded (Figure 1). In all, 72% (6943/9599) of eligible participants subscribed to the program for 12 weeks, and 2656 subscribed for 52 weeks.

The characteristics of the eligible participants are outlined in Table 1. In summary, 31% (2975/9599) of participants were overweight, 61% (5866/9599) were obese, and 86% (8279/9599) were female. The mean (SD) age of participants was 35.7 (9.5) years, 85% (8022/9455) of participants were of moderate-to-high socioeconomic status (ie, scored between 5 and 10 on IRSAD), and 75% (7125/9456) were from major cities in Australia. The majority of the group reported some healthy eating habits such as eating breakfast (7052/9599 or 74%) and using low fat products (6269/9599 or 65%), but many (5098/9599 or 53%) also reported poor eating habits such as skipping meals. Most participants reported inadequate levels of physical activity at enrollment, with 51% (4875/9569) exercising less than 2 days per week.

**Table 1:** Pretreatment characteristics

Descriptor		Total	12 Weeks	52 Weeks	*P* Value
		n = 9599	n = 6943	n = 2656
**Age (years)**					
	Mean (SD)	35.7 (9.5)	35.3 (9.4)	36.7 (9.6)	< .001
	18 to 25 years, %	12.8	13.5	10.8	< .001
	25 to 35 years, %	37.4	38.6	34.5	
	35 to 45 years, %	33.2	32.3	35.6	
	45 to 55 years, %	13.2	12.6	14.7	
	55 to 65 years, %	3.1	2.8	4.0	
	65 to 75 years, %	0.4	0.4	0.4	
Female (%)		86.3	86.5	85.7	.30
**BMI (kg/m2)**					
	Mean (SD)	32.9 (6.7)	31.8 (6.1)	35.8 (7.1)	< .001
	Healthy weight, %	7.9	9.7	3.1	< .001
	Overweight, %	31.0	35.7	18.7	
	Obese, %	61.1	54.6	78.2	
**Socioeconomic status (IRSAD decile)**^a^					
	1-2, %	5.8	4.9	8.0	< .001
	3-4, %	9.4	9.1	10.3	
	5-6, %	18.2	17.4	20.2	
	7-8, %	29.3	29.5	28.7	
	9-10, %	37.4	39.1	32.8	
**Remoteness (ARIA)b**					
	Major city, %	75.4	76.4	72.7	.001
	Regional, %	23.2	22.3	25.8	
	Remote, %	1.4	1.3	1.6	
**Days of planned exercise**^c^					
	0-1 days, %	51.0	50.6	51.8	< .001
	2 or more days, %	49.0	49.4	48.2	
**Eating habits**					
	Fry foods, %	37.9	36.4	42.4	< .001
	Use butter in cooking, %	36.1	35.4	38.2	.01
	Drink full sugar soft drinks, %	29.4	28.2	32.6	< .001
	Skip meals, %	53.1	51.3	58.0	< .001
	Drink tea or coffee with sugar, %	43.7	44.4	41.9	.03
	Eat breakfast, %	73.5	74.7	70.3	< .001
	Use low fat products, %	65.3	66.3	62.7	.001
	Keep snack foods in the house, %	59.8	58.9	62.1	.004
	Drink 6+ glasses of water a day, %	40.7	41.2	39.4	.10

**Reason for eating**					
	To ease emotional upset, %	56.0	55.0	58.7	.001
	For the joy of it, %	55.9	53.4	56.9	.002
	To reduce stress, %	44.6	44.0	46.1	.07
	Out of boredom, %	78.6	78.9	77.9	.26
					
	One or more health-related reasons for weight loss, %	54.7	53.2	58.9	< .001

^a^ Total n = 9455; at 12 weeks n = 6841; and at 52 weeks, n = 2614

^b^ Total n = 9456; at 12 weeks, n = 6842; and at 52 weeks, n = 2614

^c^ Total n = 9569; at 12 weeks, n = 6923; and at 52 weeks, n = 2646

Statistically significant differences in pretreatment characteristics of 12- and 52 week subscribers were evident, with the mean (SD) age of participants who subscribed for 52 weeks being significantly greater (35.8 [7.1] years of age vs 31.8 [6.1] years of age), having a higher mean (SD) BMI (36.7 [9.6] vs 35.3 [9.4]), being of lower socioeconomic status (82% vs 86% with an ISRAD of 5 to 10), and a lower proportion residing in major cities of Australia (73% vs 76%) when compared with 12 week subscribers. A significantly higher proportion of 52 week subscribers reported poor eating habits (eg, frying foods or drinking full sugar soft drinks), exercising less than 2 days per week, eating for emotional reasons or for the joy of it, and having health-related reasons for wanting to lose weight.

**Figure 2:**
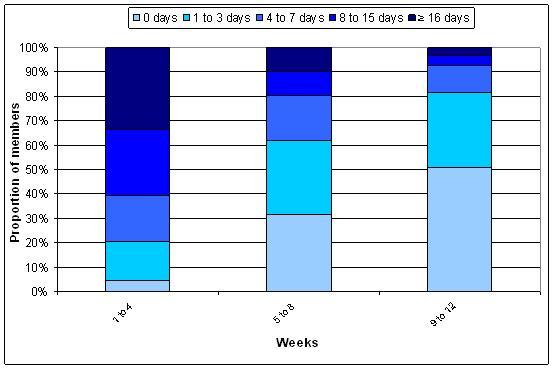
Website use from enrollment to 12 weeks among 12 week subscribers

Figures 2 describe overall website use. For both 12- and 52 week subscribers, the highest proportion of participants used the website on 16 days or more during weeks 1 to 4 of the program. During weeks 5 to 8 and weeks 9 to 12, the highest proportion of 12 week subscribers did not use the website. However, of the participants who did use the website, most used it 1 to 3 days during each 4 week period. For 52 week subscribers, the highest proportion of participants used the program on 1 to 3 days from weeks 5 to 8. After this time (ie, weeks 9 to 52) the highest proportion of participants never used the website, and the second highest proportion used the website 1 to 3 days in each 4 week period.

**Figure 3:**
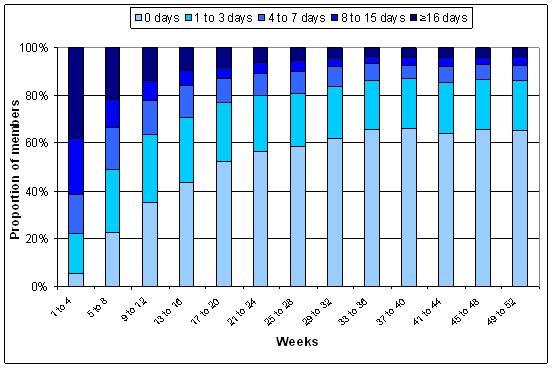
Website use from enrollment to 52 weeks among 52 week subscribers

### Dropout Attrition

**Figure 4:**
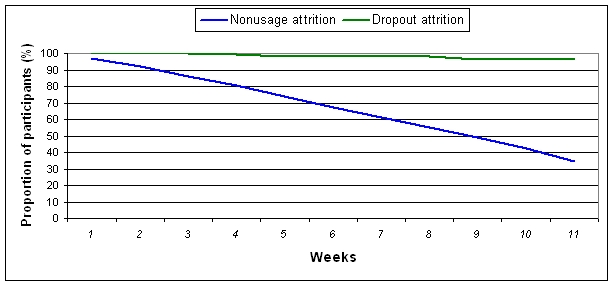
Dropout attrition and nonusage attrition from enrollment to 12 weeks among 12 week subscribers

**Figure 5:**
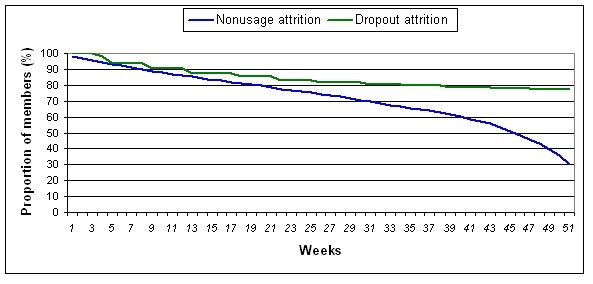
Dropout and nonusage attrition from enrollment to 52 weeks among 52 week subscribers

Figures 4 and 5 present dropout attrition curves for 12- and 52 week subscribers respectively. Of the 6943 participants who subscribed to the program for 12 weeks, the retention rate was 97% at 12 weeks, with 238 participants (3%) dropping out over the 12 week period (Figure 4). For the 2656 participants who subscribed to the program for 52 weeks, the retention rate was 77% with 605 dropping out over the 52 week period (Figure 5).

### Nonusage Attrition

Figures 4 and 5 also present nonusage attrition curves for those who subscribed and completed a 12- or 52 week subscription, respectively. Of the 6705 participants who subscribed to and completed 12 weeks of the program, 35% (2317) of participants were classified as ”users” of the program at 12 weeks. The lowest proportion of participants stopped using the program during weeks 1 and 2. The proportion of participants who stopped using the program remained steady from week 3 to week 10 (6% to 7% stopped using per week) but increased during week 11 to 8%. Of the 6705 12 week subscribers, 50% (n = 3398) had become nonusers of the program by week 9 (Figure 4).

Of the 2051 participants who completed their 52 week subscription, 622 participants (30%) were “users” of the program at 52 weeks. The proportion of participants who stopped using the program remained steady from week 1 to week 44 (1% to 2% stopped using per week) but increased rapidly thereafter. By week 46, greater than 50% of the 52 week subscribers were nonusers of the program (Figure 5).

### Predictors of Nonusage Attrition: 12 week Subscribers

Table 2 describes unadjusted predictors of nonusage attrition among 12 week subscribers from univariate analyses. In the multiple regression analysis (Table 2), skipping meals (hazard ratio = 1.12, 95% confidence interval (CI) 1.04 - 1.19, *P* = .001) and eating to ease emotional upset (hazard ratio = 1.11, 95% CI 1.04 -1.18, *P* = .001) were the 2 pretreatment characteristics found to significantly increase a participants risk of being a nonuser. Participants who exercised more than 1 day per week were at a significantly decreased risk of being a nonuser (hazard ratio = 0.76, 95% CI 0.72 - 0.81, *P* < .001). Participants who ate breakfast (hazard ratio = 0.88, 95% CI 0.82 - 0.95, *P* = .001) were also at decreased risk of nonusage, as well as participants aged 45 to 65 years (hazard ratio for 45 to 55 years of age = 0.83, 95% CI 0.73 - 0.93, *P* = .001; hazard ratio for 55 to 65 years of age = 0.80, 95% CI 0.66 - 0.99, *P* = .04).

**Table 2:** Risk of nonusage attrition for 12 week subscribers

Risk Factor		Unadjusted (n = 6705)		Adjusted (n = 6686)^d^		
		Hazard Ratio (95% CI)	*P*	Hazard Ratio (95% CI)	*P*
**Gender**						
	Male	1.00 (reference)			
	Female	0.85 (0.78 - 0.92)	< .001		
**Age (years)**						
	18 to 25	1.00 (reference)		1.00 (reference)	
	25 to 35	0.92 (0.84 -1.01)	.09	0.93 (0.85 - 1.02)	.12
	35 to 45	0.92 (0.84 -1.01)	.09	0.93 (0.85 - 1.03)	.15
	45 to 55	0.81 (0.72 - 0.91)	< .001	0.83 (0.73 - 0.93)	.001
	55 to 65	0.77 (0.63 - 0.95)	.01	0.80 (0.66 - 0.99)	.04
	65 to 75	0.54 (0.29 - 1.01)	.05	0.63 (0.34 - 1.17)	.14
**Socioeconomic status (IRSAD decile)**^a^						
	1-2	1.00 (reference)			
	3-4	1.04 (0.88 - 1.23)	.67		
	5-6	0.97 (0.83 - 1.13)	.69		
	7-8	1.01 (0.87 - 1.17)	.93		
	9-10	1.03 (0.89 - 1.19)	.72		
**Remoteness**^b^						
	Major cities of Australia	1.00 (reference)			
	Regional Australia	0.97 (0.90 - 1.04)	.35		
	Rural/remote Australia	1.17 (0.91 - 1.49)	.21		
**BMI**						
	Healthy weight	1.00 (reference)			
	Overweight	1.02 (0.92 - 1.14)	.66		
	Obese	1.11 (1.00 - 1.24)	.04		
	0 to 1 days	1.00 (reference)		1.00 (reference)	
	2 or more days	0.74 (0.69 - 0.78)	< .001	0.76 (0.72 - 0.81)	< .001
**Reason for eating**					
	To ease emotional upset	1.07 (1.01 - 1.14)	.01	1.11 (1.04 - 1.18)	.001
	For the joy of it	0.99 (0.93 - 1.05)	.63		
	To reduce stress	1.10 (1.03 - 1.16)	.002		
	Out of boredom	0.98 (0.91 - 1.05)	.59		

**Eating habits**					
	Fry foods	1.07 (0.99 - 1.13)	.07		
	Use butter in cooking	1.06 (0.99 - 1.13)	.07		
	Drink full sugar soft drinks	1.16 (1.09 - 1.24)	< .001		
	Skip meals	1.23 (1.16 - 1.31)	< .001	1.12 (1.04 - 1.19)	.001
	Drink tea or coffee with sugar	0.99 (0.94 - 1.05)	.84		
	Eat breakfast	0.77 (0.72 - 0.82)	< .001	0.88 (0.82 - 0.95)	.001
	Use low fat products	0.85 (0.79 - 0.90)	< .001		
	Keep snack foods in the house	1.03 (0.97 - 1.09)	.33		
	Drink 6 or more glasses of water a day	0.92 (0.86 - 0.97)	.004		
					
	1 or more health-related reasons for weight loss	0.97 (0.92 - 1.03)	.37		

^a^ n = 6610

^b^ n = 6611

^c^ n = 6686 (all unadjusted)

^d^ Stratified by gender

### Predictors of Nonusage Attrition: 52 week Subscribers

Table 3 describes unadjusted potential predictors of nonusage attrition for 52 week subscribers using univariate analyses. In the multiple regression analysis (Table 3), eating breakfast (hazard ratio = 0.88, 95% CI 0.79 - 0.99, *P* = .04) was shown to be associated with reduced risk of nonusage attrition. Drinking tea or coffee with sugar was associated with increased risk of nonusage attrition among 52 week subscribers (hazard ratio = 1.23, 95% CI 1.11- 1.37, *P* < .001).

**Table 3:** Risk of nonusage attrition for 52 week subscribers

Risk Factors		Unadjusted (n = 2051)		Adjusted (n = 2043)^d^	
		Hazard Ratio (95% CI)	*P*	Hazard Ratio (95% CI)	*P*	
**Gender**					
	Male	1.00 (reference)			
	Female	0.90 (0.78 - 1.04)			
**Age (years)**					
	18 to 25	1.00 (reference)			
	25 to 35	0.96 (0.79 - 1.16)	.66		
	35 to 45	0.93 (0.77 - 1.16)	.45		
	45 to 55	0.79 (0.63 - 0.97)	.03		
	55 to 65	0.68 (0.49 - 0.91)	.01		
	65 to 75	0.20 (0.02 - 1.44)	.11		
**Socioeconomic status (IRSAD decile)**^a^					
	1-2	1.00 (reference)			
	3-4	0.92 (0.71 - 1.18)	.49		
	5-6	0.82 (0.66 - 1.03)	.08		
	7-8	0.89 (0.72 - 1.10)	.29		
	9-10	0.82 (0.66 - 1.01)	.06		
**Remoteness**^b^					
	Major cities of Australia	1.00 (reference)			
	Regional Australia	1.03 (0.91 - 1.16)	.66		
	Rural/remote Australia	1.05 (0.64 - 1.71)	.89		
**BMI**					
	Healthy weight	1.00 (reference)			
	Overweight	1.02 (0.74 - 1.42)	.89		
	Obese	1.04 (0.76 - 1.41)	.83		
**Exercise level**^c^										
	0 to 1 day	1.00 (reference)			
	2 or more days	0.70 (0.63 - 0.78)	< .001		
**Reason for eating**					
	To ease emotional upset	0.98 (0.88 - 1.08)	.64		
	For the joy of it	0.96 (0.87 - 1.07)	.46		
	To reduce stress	0.93 (0.84 - 1.03)	.17		
	Out of boredom	0.97 (0.86 - 1.10)	.62		

**Eating habits**					
	Fry foods	1.12 (1.00 - 1.24)	.04		
	Use butter in cooking	1.16 (1.04 - 1.29)	.007		
	Drink full sugar soft drinks	1.12 (1.01 - 1.26)	.04		
	Skip meals	1.22 (1.10 - 1.36)	< .001		
	Drink tea or coffee with sugar	1.25 (1.13 - 1.39)	< .001	1.23 (1.11 - 1.37)	< .001
	Eat breakfast	0.82 (0.73 - 0.92)	.001	0.88 (0.79 - 0.99)	.04
	Use low fat products	0.84 (0.75 - 0.93)	.001		
	Keep snack foods in the house	1.09 (0.98 - 1.21)	.13		
	Drink 6 or more glasses of water a day	0.93 (0.83 - 1.03)	.15		
1 or more health-related reasons for weight loss		0.90 (0.81 - 1.01)	.06		

^a^ n = 2019

^b^ n = 2019

^c^ n = 2043 (all unadjusted)

^d^ Stratified by exercise level

## Discussion

This study is one of only a small number of studies [29,35-42] to follow a group of real-world participants of a Web-based weight loss program and is the first to comprehensively evaluate the prevalence and predictors of nonusage attrition in a large cohort. The study demonstrates a high prevalence of nonusage attrition and highlights the need for evidence-based strategies to reduce attrition rates. Notably, we found that a participant’s age, as well as his or her eating and physical activity habits at enrollment can predict nonusage attrition.

The findings from this study are consistent with other studies that have demonstrated that individuals in the mid-to-older age group (45 to 65 years) are at decreased risk of nonusage [13,29,30]. People in this age group have lower levels of Internet access [43], spend less time using the Internet and are less likely to use user-generated sites than younger age groups [43,44]. However, their access and use of the Internet is increasing rapidly [43,44]. Therefore, this suggests that Web-based interventions may be well suited to the mid-to-older age groups.

The study findings suggest that people with poor eating or physical activity habits prior to enrolling in a commercial Web-based weight loss program are most likely to stop using the program. This includes participants who exercised less than 2 days per week, skipped meals, did not eat breakfast, drank tea or coffee with added sugar, or identified eating to ease emotional upset. This suggests that these at-risk individuals may require alternate or additional support to remain an active participant of Web-based programs, particularly in the short-term. Alternatively, it may be that the Web-based program in its current form did not engage this group of participants. A research priority is, therefore, to determine whether different or extra website features can improve website usage in this group of at-risk individuals.

This study highlights the importance of investigating nonusage attrition to accurately describe attrition rates. The retention rates for the commercial Web-based weight loss program of 97% after 12 weeks and 77% after 52 weeks were high in comparison with observational [29,37,40,41] and experimental [5] Web-based weight loss intervention studies, as well as all types of behavioral weight loss interventions [45]. However, as participants purchase a specific subscription plan and can only unsubscribe if they have special circumstances that prevent them from completing their subscription, the retention rates do not capture those participants who did not wish to continue using the program. The nonusage attrition at 12 weeks of 65% and at 52 weeks of 70% is higher than the dropout attrition rates and demonstrates that a number of participants do not continue to use the commercial Web-based weight loss program for the duration of their subscription. Use of the commercial website was consistent with other public health interventions delivered via the Internet, whereby use drops after the preliminary weeks of the intervention [17]. For both 12- and 52 week subscribers the nonusage attrition was steady throughout the majority of the intervention, but nonusage attrition increased slightly towards the final weeks of the intervention. This opposes a previous hypothesis that suggests that by the final phase of the intervention a stable user group should exist, resulting in less nonusage [4]. The pattern of nonusage attrition in this study is most likely an interplay of several factors that potentially impact nonusage attrition either positively (eg, cost of program, program features, and usability) or negatively (eg, no prompts or personal contact, self-directed nature) [4].

To our knowledge, only 2 other studies have investigated nonusage attrition rates in a Web-based interventions aiming to achieve weight loss [29,42]. The first, an observational study, described nonusage attrition rates for a physical activity focused Web-based program (MiLife) and found that 79% of participants were still using the website after 12 weeks [42]. The second study compared nonusage attrition rates among RCT and real-world participants of a Web-based intervention (Active-Online) to promote physical activity over an 18-month period. Greater than 50% of trial participants became nonusers after approximately 11 months and 1 month for the real-world participants [29]. This commercial Web-based weight loss program’s nonusage attrition rates were superior to the real-world participants of Active-Online but higher than MiLife [29,42]. However, both Active-Online and MiLife incorporated strategies that have been previously proposed as factors that influence nonusage attrition [4]. One intervention was worksite based [42], which may have enhanced the networking and/or peer pressure and peer support, and, therefore, reversed the usual decline in nonusage [4]. A number of “push-factors” [4], including reminder emails and short message service (SMS) were utilized [29,42]; therefore, participants may have felt obligated to continue using the Web-based programs [4]. The use of accelerometers by participants in one of the studies to monitor physical activity levels [42] may have improved the usability of the program and, therefore, increased usage rates [4]. In comparison, the commercial Web-based weight loss program is primarily a self-directed intervention. This may have negatively impacted usage rates, as it made it easier for participants to stop using the program [4]. However, the participants paid a commercial rate to access the program, which has been previously suggested to positively impact usage rates [4]. As the cost of the program varied and was dependant on the length of subscription and whether a participant paid up front or in installments, the impact on nonusage may have varied. Therefore, the nonusage attrition rates reported in this study appear acceptable compared with previous studies, taking into consideration the existence of factors that may have impacted nonusage attrition.

### Limitations

Potential limitations of this study include that only pretreatment characteristics were considered as potential predictors of nonusage attrition. It is possible that other factors such as satisfaction with the program, initial and ongoing weight loss, and external factors also influenced program use. However, the aim of this study was to determine whether it is possible to predict who will use the program at enrollment. Furthermore, although a large number of pretreatment characteristics were explored as potential predictors of nonusage attrition, the study could have been improved by including a larger range of pretreatment characteristics (eg, motivation and stage of change), as well as through the use of validated measures to more comprehensively assess eating and physical activity behaviors. In addition, the study did not track the use of all features of the commercial Web-based weight loss program (eg, weekly tutorials and menu plans), as these data were not available at the time of the study. This may have overestimated nonusage attrition rates. Furthermore, the methodology assumes that nonusage is a negative behavior. It has been suggested, however, that participants may consider Web-based interventions differently from other treatment options [3]. Participants who stop using the website may have achieved a positive outcome and, therefore, reduced the frequency with which they engage with the Web-based program [3]. Further research investigating participants’ reasons for dropout and nonusage attrition and the impact of dropout and nonusage attrition on long-term weight loss is therefore required.

### Implications

Adherence has been acknowledged as one of the main determinants of effectiveness [46]; therefore, strategies are required to improve nonusage attrition rates among Web-based weight loss program participants. Previous research [5], including research with this cohort [47], has demonstrated a significant correlation between the use of different website features (eg, log-ins, use of discussion forums, online diary entries, and self-monitoring of weight) and weight change. Therefore, there is potential to improve weight loss achieved by participants of Web-based weight loss programs by establishing effective methods to improve nonusage attrition, so that the majority of participants continue to use the website features in the long-term. As the mean weight change achieved by participants of this Web-based weight loss program after 12 and 52 weeks has been found to be clinically important and statistically significant [48,49], if strategies were successful in improving engagement, the public health impact could be substantial.

The findings from this study also highlight key pretreatment sociodemographic and behavioral predictors of nonusage attrition. The findings are similar to other weight loss [21,26-28] and Web-based intervention studies [30], whereby individuals most in need of treatment are less likely to complete and/or engage with the intervention. A number of previous Web-based intervention studies have investigated Web and non-Web-based strategies to improve website engagement including periodic prompts, incentives, self-monitoring, management of participant expectations, improving intervention usability, provision of feedback, as well as contact with service providers [2]. Given the self-directed nature of this intervention, the findings suggest clear evidence-based guidelines outlining the website use required to achieve significant outcomes may also improve nonusage attrition rates. One or a combination of these strategies could be provided to the participants who enroll in the program with the pretreatment characteristics predictive of attrition. However, we do not know the most appropriate strategy or combination of strategies required to improve the use of Web-based programs or whether the strategies required are consistent across population groups. In the future, such knowledge may be used as part of the enrollment process to ensure individuals enroll who are best suited to this approach and that they are provided with access to program features within the Web-based program that meets their needs. Therefore, a research priority is the development and evaluation of strategies to improve nonusage attrition rates in Web-based programs, including their impact on different population groups.

### Conclusion

Previous research has identified optimization of participant retention and website use as key challenges for all Web-based interventions [2-4]. This study demonstrated the high prevalence nonusage attrition characteristic of Web-based interventions and, therefore, highlights the need for evidence-based strategies to improve website use. Researchers should investigate the use of new or additional intervention strategies among participants with the pretreatment demographic and behavioral characteristics that were found to independently predict nonusage attrition in this study.

## Abbreviations

BMI: body mass index
ISRAD: Index of Relative of Socioeconomic Advantage and Disadvantage
RCT: randomized controlled trial
SMS: short message service
